# Clinical and Optical Coherence Tomography Angiographic Features in Patients with Postcataract *Stenotrophomonas maltophilia* Endophthalmitis

**DOI:** 10.1155/2020/8723241

**Published:** 2020-07-04

**Authors:** Lu Chen, Dahui Ma, Jieting She, Miaohong Chen, Jian Zeng, Jiantao Wang, Guoming Zhang

**Affiliations:** Shenzhen Eye Hospital, Shenzhen Key Laboratory of Ophthalmology, 18 Zetian Road, Shenzhen, China

## Abstract

**Purpose:**

To report the clinical presentations and optical coherence tomography (OCT) angiographic features of patients with postcataract surgery endophthalmitis due to *Stenotrophomonas maltophilia. Methods*. A retrospective observational study of 4 patients who developed *S. maltophilia* endophthalmitis after cataract surgery. Pars plana vitrectomy (PPV) was performed to control the infection. Patients were followed up for six months. Complete ophthalmological examination results were collected before and after PPV.

**Results:**

Patients' response to PPV therapy was excellent and the infection was cured in all cases. OCTA showed that, at the one-month follow-up, the vascular density (VD) and perfusion density (PD) in the superficial capillary plexus (SCP) were significantly lower than those in healthy collateral eyes. As time went on, the SCP-VD and SCP-PD values gradually improved.

**Conclusions:**

With early PPV, the infection caused by *S. maltophilia* can be cured. OCTA provides a quantitative noninvasive assessment to evaluate the severity and prognosis of patients with *S. maltophilia* endophthalmitis.

## 1. Introduction

Acute postoperative endophthalmitis (POE) is an infrequent but devastating complication of cataract surgery, with an overall incidence ranging from 0.03% to 0.2% worldwide [1, 2]. Most cases of acute POE are thought to be the result of the bacterial flora that exist in the patient's own conjunctiva. According to microbiologic analyses, a large proportion of intraocular specimens from eyes with culture-proven acute POE are Gram-positive bacteria, including coagulase-negative *Staphylococcus* (except *S. lugdunensis*), *Streptococci spp.*, and *Staphylococcus aureus* [3, 4]. Gram-negative bacteria only account for approximately 4.5% of eyes with clinical evidence of acute POE [5]. However, outbreaks of POE are usually due to Gram-negative organisms [6].

*Stenotrophomonas maltophilia* (*S. maltophilia*) is an aerobic opportunistic Gram-negative bacillus bacterium that is common in aquatic environments and in soil [7]. Although it is rarely associated with human infections, nosocomial colonization is the primary cause of infection by this bacillus [8]. Ophthalmic infections of *S. maltophilia* have been reported to include keratitis [9], preseptal cellulitis, and infantile dacryocystitis [10]; however, few cases of *S. maltophilia* endophthalmitis after cataract surgery have been reported in the literature [8, 11, 12]. Additionally, these previous reports mainly focused on the clinical features, predisposing factors, antimicrobial susceptibility, and prognosis. To date, there have been no retrospective or prospective studies regarding the microvascular characteristics of postcataract *S. maltophilia* endophthalmitis. Optical coherence tomography angiography (OCTA) is an innovative tool that provides depth-resolved imaging of the retinal and choroidal microvasculature [13]. Thus, additional data are needed to better understand the microvasculature alterations associated with *S. maltophilia* endophthalmitis.

Herein, we reported 4 cases of *S. maltophilia* endophthalmitis after clear corneal cataract surgery evaluated for clinical features, treatments, outcomes, and OCTA features. The results might contribute to an in-depth understanding of the mechanism and treatment options for *S. maltophilia* endophthalmitis.

## 2. Methods

In this retrospective observational study, 4 patients who had *S. maltophilia* endophthalmitis after cataract surgeries were included between March 2019 and November 2019. The study was approved by the Institutional Review Board of Shenzhen Eye Hospital (Shenzhen China) and was conducted in accordance with the World Medical Association Declaration of Helsinki. Oral informed consent was obtained from each patient.

Following the guidelines of the Endophthalmitis Vitrectomy Study (EVS) [5], the 4 patients who had uneventful cataract surgeries underwent immediate pars plana vitrectomy (PPV) due to acute postcataract endophthalmitis at the discretion of one experienced surgeon. Cultures of the vitreous from each patient were positive for *S. maltophilia*. Preoperative BCVAs were determined.

The patients were scheduled for follow-up on days 1 and 7 and at 1-, 2-, 3-, and 6-month postoperative intervals. At each follow-up, patients were examined for BCVA, tonometry, slit-lamp biomicroscopy, fundus examination, OCT (Carl Zeiss Meditec, Inc., Dublin, CA, USA), and OCTA (AngioPlex, Zeiss Meditec, Inc., Dublin, CA, USA). The follow-up period of the patients with eye complications ranged from 160 to 174 days.

**Figure 1:**
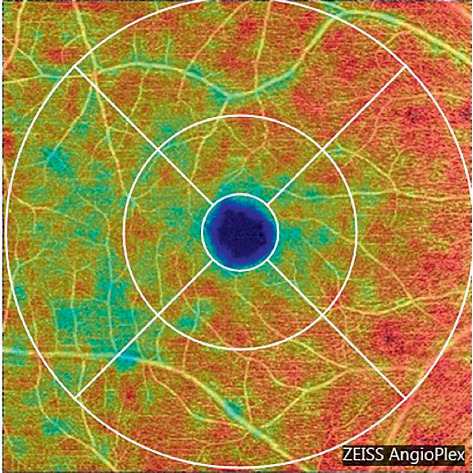
Illustration of representative 6 × 6 mm macular OCTA in SCP with false colors and ETDRS subfield analysis. CF, central foveal; IA, inner average; II, inferior inner; IO, inferior outer; NI, nasal inner; NO, nasal outer; OA, outer average; SI, superior inner; SO, superior outer; TI, temporal inner; TO, temporal outer; OCTA, optical coherence tomography angiography; SCP, superficial capillary plexus; ETDRS, Early Treatment Diabetic Retinopathy Study.

OCTA parameters, including the vascular density (VD) and perfusion density (PD) of the superficial vascular plexus (SVP), were quantified from the 6.0 × 6.0 mm cube scan centered on the fovea. The SCP refers to the vascular network that encompasses the retinal nerve fibers and ganglion cell layers. The macular scanning area was segmented based on the Early Treatment Diabetic Retinopathy Study (ETDRS) into macular circles and grids [14] and 9 subfields: central foveal, inferior inner, inferior outer, nasal inner, nasal outer, superior inner, superior outer, temporal inner, and temporal outer quadrants (Figure 1).

## 3. Results

The 4 uneventful phacoemulsification surgeries were performed between March 19, 2019, and March 26, 2019, by 4 experienced surgeons. The predominant surgical steps included topical anesthesia, temporal clear corneal incision, continuous circular capsulorhexis, and uncomplicated phacoemulsification with foldable in-the-bag intraocular lens (IOL) implantation. Cefuroxime was the intracameral antibiotic used for all patients.

**Figure 2:**
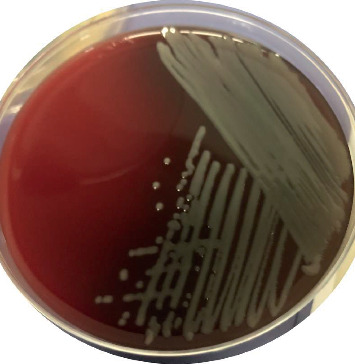
Blood agar plate culture positive of *Stenotrophomonas maltophilia*. Microbiological examination of the vitreous sample from Case 2 revealed the culture growth of *Stenotrophomonas maltophilia* on blood agar.

However, a few days postoperatively (average 11 days, ranging from 9 to 12 days), all these patients presented with sudden onset of reduced vision and increasing eye pain and were diagnosed with acute endophthalmitis. The clinical presentations and outcomes are summarized in Table 1. Immediate PPV and aqueous or vitreous culture and sensitivity assessments were carried out, and *S. maltophilia* was isolated (Figure 2).  Table 2 summarizes the details of the treatment of these patient eyes. Systemic and topical antibiotics were used according to the sensitivity results (Table 3).

Case 2 was described in detail as a representative example of overall cases. Case 4 was described since the patient had undergone operation twice to control the infection.

### 3.1. Case 2

A woman in her 70s underwent clear cornea phacoemulsification and IOL implantation with no complications. The ophthalmic, medical, and systemic histories were unremarkable. Seven days after cataract surgery, she achieved a BCVA of 0.15 (logMAR) in the eye. Unfortunately, 12 days postoperatively, she experienced severe acute pain in the affected eye. Visual acuity decreased to only hand motion at a distance of 10 cm. Slit-lamp and fundus examinations showed hypopyon, fibrin, marked anterior chamber reaction, vitreous cells and haze, loss of the red reflex, and RAPD (+). No view of the retina was available. She was diagnosed with acute postoperative endophthalmitis, and immediate PPV was carried out with the intravitreal injection of vancomycin and silicone oil tamponade. During the operation, the IOL and capsule bag were removed. Vitreous culture proved to be positive for *S. maltophilia*. According to the antibiotic sensitivity results, systemic gentamicin (160 mg) was used for three days accompanied by topical levofloxacin/ciprofloxacin/prednisolone acetate q1h with gradual tapering. The patient responded extremely well, and the BCVA improved to 0.15 (logMAR) within a 6-month period. At that time, the anterior chamber and the vitreous humor were clear, and no other signs of inflammation were noted. No recurrence was found.

### 3.2. Case 4

**Figure 3:**
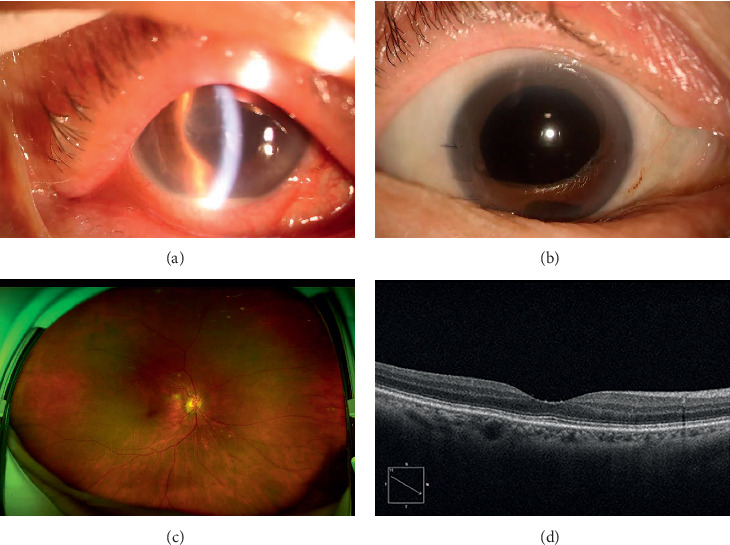
Typical figures of *Stenotrophomonas maltophilia* endophthalmitis at onset and after PPV, IOL removal, and silicone oil tamponade. (a) Fibrin and hypopyon were found in the anterior chamber 12 days following cataract surgery in Case 2. The corneal edema and Descemet's membrane striae were also present. (b) The same patient six months after PPV surgery. The anterior chamber was clear and the anterior segment was quiet. (c) SLO examination showed that the fundus was flat. The optic disc and vessels are normal. (d) OCT examination showed that the ultrastructure of macular was relatedly normal. The layers of macular could be clearly seen. IOL, intraocular lens; PPV, pars plana vitrectomy; SLO, scanning laser ophthalmoscopy.

A 67-year-old female complained of acute pain and dramatic decrease of vision in her right eye. Eleven days ago, she had an uneventful phacoemulsification and IOL implantation of this eye. She had a systemic history of high blood pressure, metrocarcinoma, and hyperthyroidism. At onset, BCVA was FC/40 cm in her right eye. Slit-lamp examination showed pupillary membrane, hypopyon, and RAPD (+) (Figure 3(a)). Dense vitreous opacities could also be observed using slit lamp. Red reflex was lost. B scan demonstrated vitreous echoes (++). According to the clinical findings, acute postoperative endophthalmitis was confirmed. PPV and intravitreal injection of vancomycin were performed. However, the IOL was not removed and silicone oil was not used neither. *S. maltophilia* was found in the aqueous humor. Systemic gentamicin (160 mg) and tobramycin were used according to the antibiotic sensitivity results. But five days after the surgery, there were still plenty of fibrin exudates in the anterior chamber and dense vitreous opacities. Therefore, the patient was given a second procedure with IOL removal and silicone oil tamponade. Fortunately, the infection was cured. At 6-month follow-up, the anterior segment was quiet, the retina looked normal, and OCT scan showed the microstructure of macular was unremarkable (Figures 3(b)–3(d)). BCVA improved to 0.52 (logMAR). There were no signs of recurrence.

### 3.3. Overview of Case Findings during Follow-Up

The conditions of these 4 patients were stable after discharge without recurrence. LogMar BCVAs were 0.15, 0.05, 0.92, and 0.52, respectively, at 6-month follow-up (Table 1).

**Figure 4:**
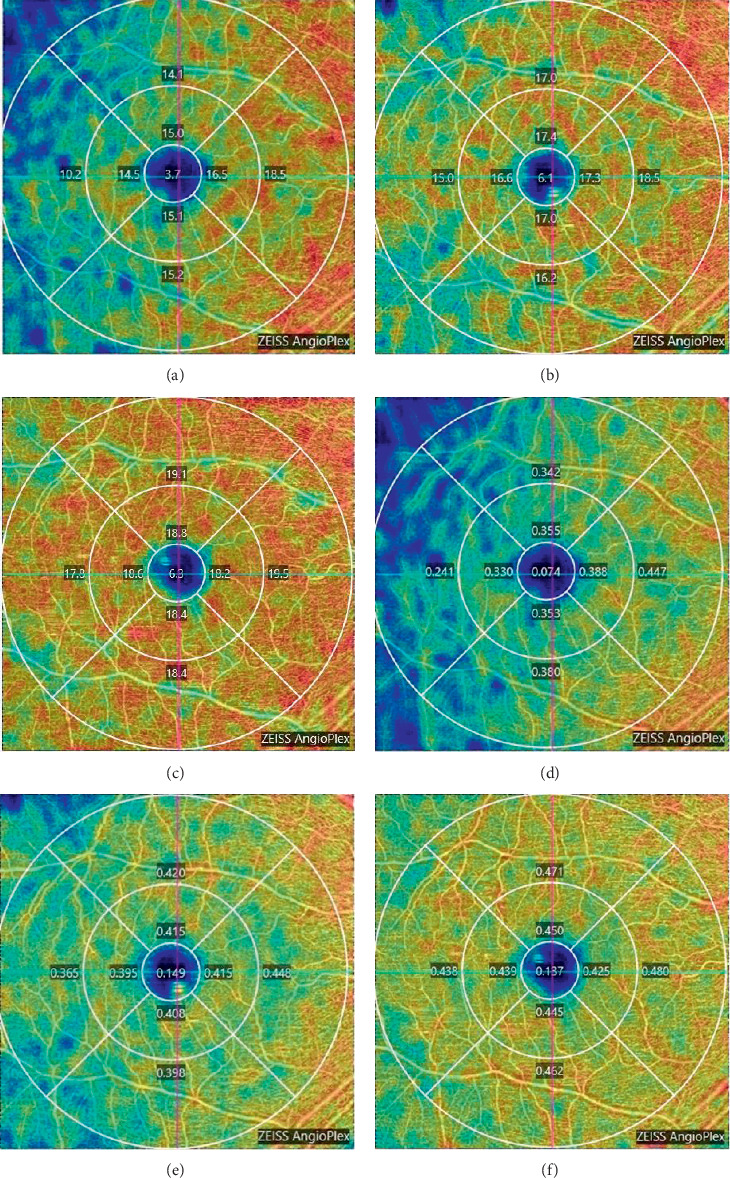
Typical figures of OCTA evaluation during one-month, three-month, and six-month follow-up. (a–c) The vessel diameter maps for three visits. (d–f) The perfusion density maps for all three visits in Case 1. The highest-density areas are highlighted in red and orange while the lowest-density areas are shown in blue and teal. The VD and PD of each ETDRS subfield gradually increased during six month's follow-up. OCTA, optical coherence tomography angiography; VD, vascular density; PD, perfusion density; ETDRS, Early Treatment Diabetic Retinopathy Study.

At the one-month follow-up, the VD-SVP and PD-SVP in both the foveal and parafoveal areas and every subfield were lower in the postoperative eyes relative to the healthy collateral eyes. During the 3- and 6-month follow-up periods, both the VD-SVP and PD-SVP values increased (see Supplementary Tables 1 and 2 in the Supplementary Material for the changes of vascular density and perfusion density) (Figure 4).

## 4. Discussion

The current study described the clinical characteristics, final outcomes, and, most importantly, microvascular alterations in 4 patients with *S. maltophilia* endophthalmitis with the use of OCTA.

Currently regarded as a rare community-acquired infection, *S. maltophilia* has gained recognition as an important nosocomial pathogen [15]. Unfortunately, this pathogen is resistant to numerous antibiotics, leading to clinical virulence of the infection. Since it was first described in 1989 [16], *S. maltophilia* endophthalmitis has been sporadically reported in a very limited number of case reports and in small case series [17–19].

The average latency of the isolates in our study was 11 days (ranging from 9 to 12 days), which was in accordance with a previous report (13.5 days) [11]. However, the results of various other studies differ. Kaiser et al. [17] reported a case of *S. maltophilia* endophthalmitis 6 days after cataract surgery. In another study, two patients with diabetes mellitus developed *S. maltophilia* endophthalmitis within 5 days after IOL implantation [20]. This might be due to variations in the virulence of *S. maltophilia* and to the different immune statuses of patients.

*S. maltophilia* is not a normal flora in the conjunctiva or periocular skin. It might cause exogenous endophthalmitis after cataract surgeries and endogenous endophthalmitis in immunocompromised or hospitalized patients [21]. Because of its frequent affinity for water sources, the nosocomial origin has been reported to be from a resterilized aspiration tube [11], the aspiration fluid of the internal vacuum control manifold (VCM) of phaco machines [22], a phacoemulsifier [19], or ophthalmic solutions (e.g., balanced salt solution) [23]. In our series, although the source of infection was not identified in any of the hospital instruments or fluids, these 4 surgeries were performed in the same operating room with the same phaco machine. This situation alerted staff in the hospital to the need for much more strict sterilization of these instruments and environmental materials.

The clinical characteristics of our patients were similar to those of other documented cases [18]. The onset of the anterior chamber and vitreous infections was acute and severe. However, a significant issue was that all cases of endophthalmitis were cured after early PPV, except for Case 4. In Case 4, PPV was initially carried out with the preservation of the IOL, but the intraocular inflammation worsened. Thus, a second PPV was performed with the IOL, bag removal, and silicone oil tamponade. Therefore, this result could infer that the capsule bag and IOL might be important places where *S. maltophilia* organisms prefer to stay and that IOL removal may be crucial for the resolution of endophthalmitis. The benefits of vitrectomy might be due to the mechanical removal of *S. maltophilia* and toxins from the eye.

Fundus examination with indirect ophthalmoscopy or photography could not explain how and to what extent *S. maltophilia* affected the structure and function of the eyes. With the use of OCTA, quantitative measurements of retinal blood vessels down to the capillary level were available [24].

The patients described in our study demonstrated decreased SCP-VD and SCP-PD values at the one-month follow-up after PPV, results that were also seen in retina ischemia, and this outcome was followed by gradually increasing SCP-VD and SCP-PD values and macular thinning. Although there have been no prior OCTA studies of *S. maltophilia* endophthalmitis patients, reports on the microvascular features of retinal vasculitis using OCTA, with a common pathogenesis to endophthalmitis, have been published [25, 26]. Crossman et al. employed wide-field OCTA in 40 eyes with intermediate uveitis and vasculitis and demonstrated hypoperfusion in the SCP. A genome analysis of *S. maltophilia* revealed the presence of secreted extracellular enzyme genes such as phospholipase C in the genome [27]. Phospholipase C plays an important role in inflammatory pathways and is implicated in virulence due to its ability to degrade cell membranes as well as toxin-induced vessel contraction. Therefore, it has been proposed that infection causes direct damage and infiltration of vessel walls, leading to dysfunction of endothelial cells and occlusion of vessels, thereby impacting the macular microvasculature. After the infection was eradicated, SCP-VD and SCP-PD values gradually increased.

There were potential limitations in the current study. First, only a small number of cases were included in the research, which may have biased our results. Second, we simply analyzed microvasculature alterations in the SCP, while the deep vascular plexus and choriocapillaris were not evaluated.

In summary, based on all the findings, we concluded that *S. maltophilia* was resistant to multiple currently used antibiotics and therefore presents a special challenge for medical staff. Early PPV combined with IOL removal played a critical role in salvaging all eyes with *S. maltophilia* endophthalmitis. OCTA, which is a valuable imaging modality, demonstrated reduced SCP-VD and SCP-PD values in eyes with *S. maltophilia* endophthalmitis. Once the infection was under control, the microvasculature status of the SCP gradually improved, while the patients gained more favorable BCVAs and had clear media and quiet anterior chambers.

## Figures and Tables

**Table 1:** Demographics and clinical characteristics of cases with postcataract *S. maltophilia* endophthalmitis.

Cases	Age (years)	Gender	Systemic conditions	PCR	Postoperative days to evaluation	Presenting BCVA	SL examination	Fundus examination	US finding	Final BCVA	Follow-up (days)
1	35	M	No	No	9	FC/40 cm	Corneal edema, hypopyon, AC cells, fibrin, RAPD (+)	Dense vitreous opacities	Vitreous echoes++	0.15	174

2	78	F	No	No	12	HM/10 cm	AC flare, AC exudates, RAPD (+)	Loss of red reflex	Vitreous echoes++ ∼+++	0.05	167

3	76	F	Encephalorrhagia	No	12	FC/10 cm	Corneal edema, AC cells, hypopyon, RAPD (+)	Loss of red reflex	Vitreous echoes ++	0.92	160

4	67	F	HBP, DM, metrocarcinoma, hyperthyroidism	No	11	FC/40 cm	Pupillary membrane, hypopyon, RAPD (+)	Dense vitreous opacities Loss of red reflex	Vitreous echoes ++	0.52	172

BCVA, best-corrected visual acuity; SL, slit lamp; US, ultrasound; FC, figure counting; HM, hand motion; AC, anterior chamber; RAPD, relative apparent pupillary defect; PCR, posterior capsule rupture; F, female; M, male; HBP, high blood pressure; DM, diabetes mellitus.

**Table 2:** Management of patients with postcataract *S. maltophilia* endophthalmitis.

Cases	Specimen	Organism identified	Treatment			Complication
			Systemic (IV)	Topical	Surgical	
1	Vitreous aspirate	*Stenotrophomonas maltophilia*	Ceftazidime Vancomycin	Pred q2h Lev q2h	PPV + IOLR + SO + IOAB	IOP elevation

2	Vitreous aspirate	*Stenotrophomonas maltophilia*	Levofloxacin Gentamicin	Pred q2h Lev q2h Cipr q2h	PPV + IOLR + SO + IOAB	No

3	Vitreous aspirate	*Stenotrophomonas maltophilia*	Tobramycin Gentamicin	Pred q2h Lev q2h Gent qid	PPV + IOLR + SO + IOAB	IOP elevation

4	AC aspirate	*Stenotrophomonas maltophilia*	Tobramycin Gentamicin	Pred q2h Lev q2h	PPV + IOAB; PPV + IOLR + SO + IOAB	No

AC, anterior chamber; Pred, prednisolone acetate; Lev, levofloxacin; Cipr, ciprofloxacin; Gent, gentamicin; Tobra, tobramycin; PPV, pars plana vitrectomy; IOLR, intraocular lens removal; SO, silicone oil; IOAB, intraocular antibiotics; IOP, intraocular pressure.

**Table 3:** Antibiotic sensitivity and resistance profile for cases of endophthalmitis caused by *Stenotrophomonas maltophilia*.

	Cases			
	1	2	3	4
Sulfamethoxazole	S	S	S	S
Amikacin	N/A	S	R	S
Ciprofloxacin	S	S	R	S
Levofloxacin	N/A	S	S	S
Gentamicin	S	S	R	S
Tobramycin	N/A	S	R	S
Imipenem	N/A	R	R	R
Ceftazidime	R	R	R	R
Cefepime	R	R	R	R
Ampicillin	N/A	R	R	R
Cefazolin	N/A	R	R	R

N/A, not available; R, resistant; S, sensitive.

## Data Availability

The data used to support the findings of this study are available within the article.

## Supplementary Materials

Supplementary Materials Supplementary Table 1: changes of vascular density at the level of the superficial capillary plexus during the follow-up after PPV. Supplementary Table 2: changes of perfusion density at the level of the superficial capillary plexus during the follow-up after PPV.
