# Supplementary material for: Clinical and Optical Coherence Tomography Angiographic Features in Patients with Postcataract Stenotrophomonas maltophilia Endophthalmitis
Source: J Ophthalmol. 2020 Jul 4;2020:8723241. doi: 10.1155/2020/8723241 (PMC7355360; doi:10.1155/2020/8723241)
Supplement: Supplementary Materials — Supplementary Table 1: changes of vascular density at the level of the superficial capillary plexus during the follow-up after PPV. Supplementary Table 2: changes of perfusion density at the level of the superficial capillary plexus during the follow-up after PPV. [file 8723241.f1.pdf]

Supplementary table 1 Changes of Vascular Density at the level of the superficial capillary plexus during the follow-up after PPV

	Case 1			Case 2			Case 3			Case 4		
	1	2	3	1	2	3	1	2	3	1	2	3
CF	7.4	13.6	15.5	7.2	12.3	13.4	4.2	5.6	7	3.7	6.2	7.2
IA	12.5	16.7	17.9	16.4	18.9	18.9	16.6	18.8	19.1	15.3	17.1	18.3
SI	15	17.5	19.2	17.5	19.7	18.7	15.8	18.9	18.9	15	17.4	17.8
NI	14.1	19.1	19.2	16.2	19.1	19.5	16.8	18.4	18.7	16.5	17.3	18.7
II	12.2	15.9	17	15.2	18.2	19.6	17	18.8	18.8	15.1	17.1	18.8
TI	8.7	14.1	16.2	16.6	18.8	17.7	17	19.2	19.8	14.5	16.6	18
OA	14.8	16.5	17.5	17.4	18.6	18.7	17.4	19.5	19.9	14.5	16.7	18.7
SO	16.4	17.7	19.2	18.1	19.2	18.8	17	18.7	19.4	14.1	17	18.9
NO	18.5	20	20.2	18.2	19.1	20.3	18.9	20.1	20.8	18.5	18.6	19.5
IO	16.4	17.5	18	15.6	18.4	19.3	17.7	19.9	19.9	15.2	16.2	19.5
TO	7.7	10.8	12.7	17.5	17.6	16.4	15.9	19.3	19.3	10.2	15	17
Total	14.1	16.5	17.5	16.9	18.5	18.6	16.8	19	19.3	14.4	16.5	18.3

CF, central foveal; IA, InnerAverage; II, inferior inner; IO, inferior outer; NI, nasal inner; NO, nasal outer; OA, OuterAverage; SI, superior inner; SO, superior outer; TI, temporal inner;TO, temporal outer

Supplementary table 2 Changes of perfusion Density at the level of the superficial capillary plexus during the follow-up after PPV

	Case 1			Case 2			Case 3			Case 4		
	1	2	3	1	2	3	1	2	3	1	2	3
CF	0.157	0.295	0.36	0.159	0.291	0.279	0.09	0.126	0.272	0.074	0.149	0.152
IA	0.287	0.389	0.42	0.398	0.451	0.462	0.392	0.456	0.458	0.357	0.408	0.438
SI	0.342	0.413	0.45	0.433	0.45	0.488	0.367	0.457	0.457	0.355	0.417	0.426
NI	0.331	0.446	0.46	0.393	0.454	0.461	0.382	0.439	0.449	0.388	0.416	0.447
II	0.28	0.379	0.41	0.365	0.473	0.448	0.412	0.457	0.47	0.353	0.406	0.458
TI	0.195	0.318	0.37	0.399	0.429	0.45	0.409	0.472	0.455	0.33	0.395	0.422
OA	0.357	0.402	0.43	0.434	0.463	0.472	0.418	0.476	0.48	0.353	0.408	0.462
SO	0.399	0.434	0.47	0.454	0.479	0.49	0.404	0.451	0.484	0.342	0.418	0.471
NO	0.449	0.499	0.51	0.461	0.495	0.482	0.46	0.487	0.499	0.447	0.45	0.478
IO	0.391	0.416	0.44	0.389	0.482	0.463	0.423	0.487	0.475	0.38	0.397	0.486
TO	0.187	0.259	0.31	0.434	0.395	0.451	0.383	0.477	0.461	0.241	0.366	0.414
Total	0.335	0.396	0.43	0.418	0.456	0.464	0.403	0.461	0.469	0.346	0.401	0.448

CF, central foveal; IA, InnerAverage; II, inferior inner; IO, inferior outer; NI, nasal inner; NO, nasal outer; OA, OuterAverage; SI, superior inner; SO, superior outer; TI, temporal inner;TO, temporal outer
